# Supplementary material for: Expression of PnSS Promotes Squalene and Oleanolic Acid (OA) Accumulation in Aralia elata via Methyl Jasmonate (MeJA) Induction
Source: Genes (Basel). 2023 May 23;14(6):1132. doi: 10.3390/genes14061132 (PMC10298014; doi:10.3390/genes14061132)
Supplement: Supplementary file 1 [file genes-14-01132-s001.zip › Supplementary Materials.pdf]

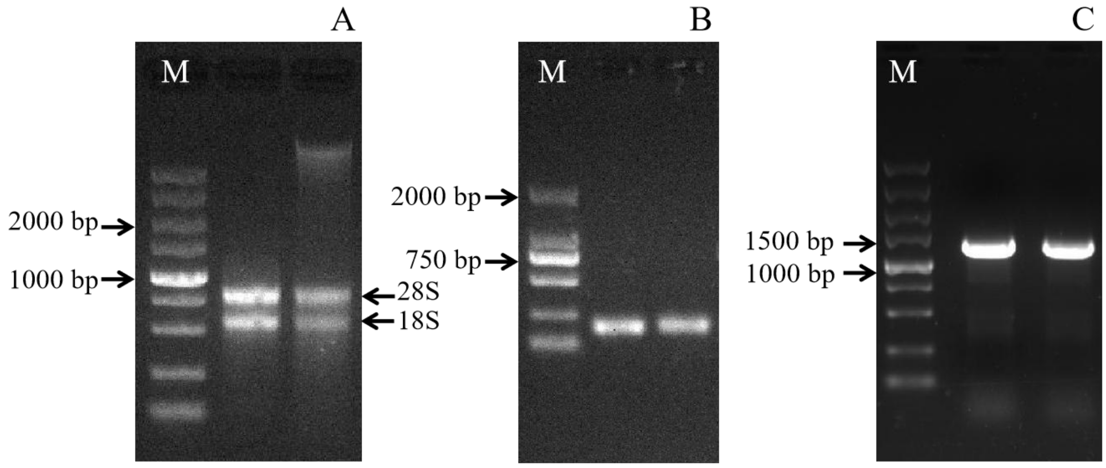

Figure S1. (A) The RNA electrophoresis, (B) quality verify of cDNA entreated from *Panax notoginseng* and (C) the electrophoretogram of PCR amplification of *PnSS* gene.
